# Supplementary material for: Three-dimensional solitary waves with electrically tunable direction of propagation in nematics
Source: Nat Commun. 2019 Aug 21;10:3749. doi: 10.1038/s41467-019-11768-8 (PMC6704189; doi:10.1038/s41467-019-11768-8)
Supplement: Supplementary file 1 — Supplementary Information [file 41467_2019_11768_MOESM1_ESM.docx]

Supplementary Information

**Three-dimensional solitary waves with electrically tunable direction of propagation in nematics**

Li et al.


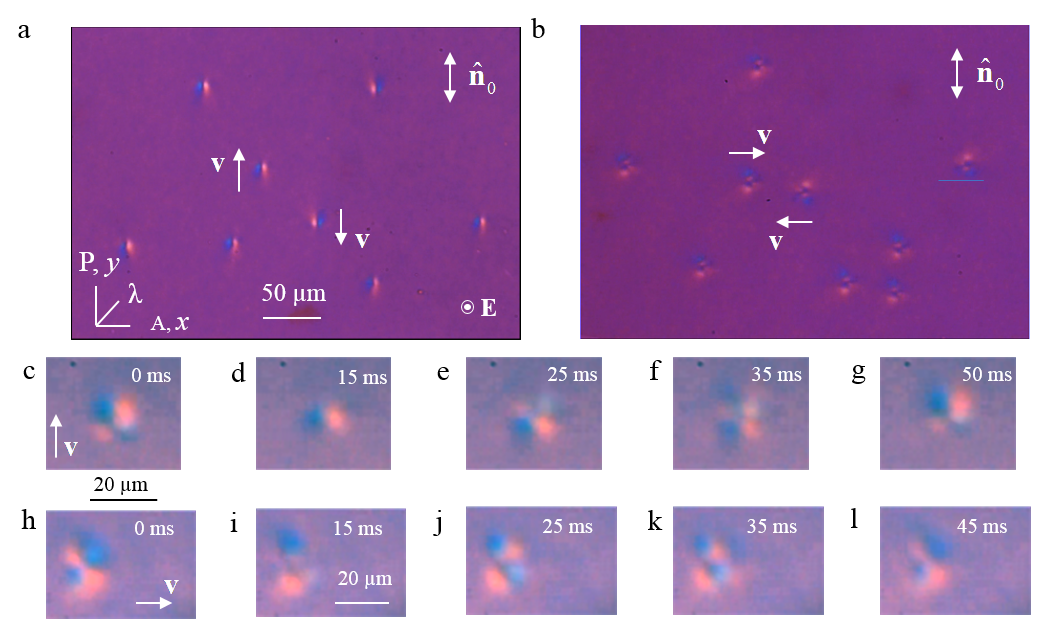


**Supplementary Fig. 1| Solitons observed between polarizers and a waveplate. a,** Solitons $\text{B}_{0}^{\text{l}}$ propagate along the director $\hat{\mathbf{n}}_{0}$ (20 Hz, 8.0 V); **b,** Solitons $\text{B}_{90}^{\text{l}}$ propagate perpendicularly to $\hat{\mathbf{n}}_{0}$ (20 Hz, 11.0 V). The electric field is normal to the *xy* plane. **c**-**g,** time sequence of polarizing microscope images of the $\text{B}_{0}^{\text{l}}$ soliton (20 Hz, 8.0 V). **h**-**l,** the same for the $\text{B}_{90}^{\text{l}}$ soliton (20 Hz, 11.0 V). Directions of the polarizer and analyser are indicated by axes P and A; λ is the slow axis of the optical compensator.
