# Supplementary material for: Three-dimensional solitary waves with electrically tunable direction of propagation in nematics
Source: Nat Commun. 2019 Aug 21;10:3749. doi: 10.1038/s41467-019-11768-8 (PMC6704189; doi:10.1038/s41467-019-11768-8)
Supplement: Supplementary file 3 — Description of Additional Supplementary Files [file 41467_2019_11768_MOESM3_ESM.docx]

**Description of Additional Supplementary Files**

**File Name: Supplementary Movie 1**

**Description:** Director bullets $\text{B}_{0}^{\text{l}}$. The cell of thickness $d=8 \text{μm}$, AC field applied normally to the plane of view; $U=8 \text{V}$, $f=20 \text{Hz}$; temperature $55^{\circ}\text{C}$. The solitons are moving parallel to the background director which is along the vertical direction and perpendicular to the electric field. Real time video rate.

**File Name: Supplementary Movie 2**

**Description:** Director bullets $\text{B}_{90}^{\text{l}}$. The cell thickness $d=8 \text{μm}$, AC field applied normally to the plane of view; $U=11 \text{V}$, $f=20 \text{Hz}$; temperature $55^{\circ}\text{C}$. The solitons are moving perpendicularly to the initial director which is along the vertical direction and perpendicularly to the applied electric field. Real time video rate.

**File Name: Supplementary Movie 3**

**Description:** Transformation of $\text{B}_{0}^{\text{l}}$into $\text{B}_{90}^{\text{l}}$. The voltage is abruptly raised from 8 V to 11 V at $t=0 \text{s}$; $f=20 \text{Hz}$, $T=55^{\circ}\text{C}$, $d=8 \text{μm}$. Real time video rate.

**File Name: Supplementary Movie 4**

**Description:** Transformation of $\text{B}_{90}^{\text{l}}$ into $\text{B}_{0}^{\text{l}}$. The voltage is abruptly decreased from 11 V to 8 V at $t=0 \text{s}$; $f=20 \text{Hz}$, $T=55^{\circ}\text{C}$, $d=8 \text{μm}$. Real time video rate.

**File Name: Supplementary Movie 5**

**Description:** Velocity reversal of $\text{B}_{90}^{\text{l}}$ soliton. The voltage is switched from 11 V to 13 V and then back to 11 V. $f=20 \text{Hz}$, $T=55^{\circ}\text{C}$, $d=8 \text{μm}$. Real time video rate.

**File Name: Supplementary Movie 6**

**Description:** Velocity reversal of $\text{B}_{0}^{\text{l}}$ soliton. The voltage is switched from 8 V to 7 V and then back to 8 V. $f=20 \text{Hz}$, $T=55^{\circ}\text{C}$, $d=8 \text{μm}$. The original video is taken at the frame rate of 60 fps. The playback speed is 20 fps.

**File Name: Supplementary Movie 7**

**Description:** Collision of $\text{B}_{0}^{\text{l}}$ solitons that produces transient $\text{B}_{90}^{\text{l}}$ solitons. $U=8 \text{V}$, $f=20 \text{Hz}$, $T=55^{\circ}\text{C}$, $d=8 \text{μm}$. Real time video rate.

**File Name: Supplementary Movie 8**

**Description:** Collision of two $\text{B}_{0}^{\text{l}}$ solitons that form a single $\text{B}_{90}^{\text{l}}$ soliton. $U=8.5 \text{V}$, $f=20 \text{Hz}$, $T=55^{\circ}\text{C}$, $d=8 \text{μm}$. Real time video rate.
